# Urgent needs for second life using and recycling design of wasted electric vehicles (EVs) lithium-ion battery: a scientometric analysis

**DOI:** 10.1007/s11356-024-33979-3

**Published:** 2024-06-19

**Authors:** Aqib Zahoor, Róbert Kun, Guozhu Mao, Ferenc Farkas, András Sápi, Zoltán Kónya

**Affiliations:** 1 School of Environmental Science and Engineering, Tianjin University, Tianjin, 300350 China; 2 National Industry-Education Platform of Energy Storage, Tianjin University, Tianjin, 300072 China; 3 Solid-State Energy Storage Research Group, Institute of Materials and Environmental Chemistry, Research Centre for Natural Sciences, Budapest, Magyar Tudósok Krt. 2, 1117 Budapest, Hungary; 4 Department of Chemical and Environmental Process Engineering, Faculty of Chemical Technology and Biotechnology, Budapest University of Technology and Economics, Műegyetem Rkp. 3, 1111 Budapest, Hungary; 5 Interdisciplinary Excellence Centre, Department of Applied and Environmental Chemistry, University of Szeged, Rerrich Béla Tér 1, 6720 Szeged, Hungary

**Keywords:** SLB and ELB use, Scientometric analysis, GHG emission, Hydrometallurgical technologies, Eco-friendly

## Abstract

Currently, lithium-ion batteries are increasingly widely used and generate waste due to the rapid development of the EV industry. Meanwhile, how to reuse “second life” and recycle “extracting of valuable metals” of these wasted EVBs has been a hot research topic. The 4810 relevant articles from SCI and SSCI Scopus databases were obtained. Scientometric analysis about second life using and recycling methodologies of wasted EVBs was conducted by VOSviewer, Pajek, and Netdraw. According to analytical results, the research of second life using and recycling mythologies has been growing and the expected achievement will continue to increase. China, Germany, the USA, Italy, and the UK are the most active countries in this field. Tsinghua University in China, “Fraunhofer ISI, Karlsruhe” in Germany, and “Polytechnic di Torino” in Italy are the most productive single and collaborative institutions. The journals *SAE technical papers* and *World Electric Vehicle Journal* have the highest publication and citations than other journals. Chinese author “Li Y” has the highest number of 36 publications, and his papers were cited 589 times by other authors. By analyzing the co-occurrence and keywords, energy analysis, second life (stationary using, small industry), and treatment methods, (hydrometallurgy and pyrometallurgical, electrochemical, bio-metallurgical) were the hot research topics. The S-curve from the article indicates hydrometallurgical and bio-metallurgical methods are attached with great potential in the near future. Further, different treatment methodologies are observed especially advanced techniques in hydrometallurgical, and spent medium bioleaching techniques in bio-metallurgical are good, economically cheap, has low CO_2_ emission, environmentally friendly, and has high recovery rate. Finally, this research provides information on second life use and top recycling methodology opportunities for future research direction for researchers and decision-makers who are interested in this research.

## Introduction

The transportation sector has been one of the highest contributors of wasted lithium-ion battery (LIBs) since the twenty-first century (Ding et al. 2019; Zahoor et al. 2023d). Several studies have shown that from 2001 to 2020, about 58% of the total wasted LIBs originated from tight vehicles such as E-cars, E-bikes, and light vehicles (Zhang et al. 2024). The demand for electric vehicles (EVs) is on the rise; hence, there will naturally be an increase in wasted LIBs in the future. The quest is to address the challenges of managing these wasted batteries in the sense of reusing or recycling them or overcoming their load on environmental pollution, economic, wealth, extraction of raw material, etc. (Zahoor et al. 2023c). By the year 2030, it is estimated that 1.6 MMT of used electric vehicle battery (EVB) packs will have been wasted; China, the USA, Canada, Australia, France, Germany, and some other European countries will be the big market of this wasted material (Zaefarian et al. 2022). Moreover, this means a source of 160,000 tons of valuable metals (lithium, cobalt, aluminum, and manganese) will be present in the form of waste material. Moreover, other components such as plastic, steel body cover, carbon, and liquids chemicals can be recovered (White and Swan 2023).

Currently, in wasted batteries, lithium-ion batteries (LIBs) are good to be reused or recycled because Li metals have good properties such as having the highest reducing potential, i.e., they are a very powerful reducing agent, has low specific gravity, provides high energy density (200 Wh/kg), has broad operating temperature (− 40 to − 70 °C), and has high lifetime as compared to other metals, which are the main reasons why Li-ion–based batteries are heavily recycled and used in batteries and lately in the EV automotive industry (Thakur et al. 2022).

The first life cycle of batteries, i.e., original usage in E-cars or EVs as grip battery, is about 8 to 10 years and almost a 160,000-km drive range or about 1000 cycles resulting suffer a 15 to 20% loss in capacity (Temporelli et al. 2020). However, 60–65% capacity remains that can no longer be used economically in E-car or EVs. Consequently, the number of batteries depleted in this way is increasing rapidly for the above reasons. After the first life cycle of these batteries, there are main two alternative possibilities (Shekhawat and Bansal 2023). Firstly, it is reused for the second life purpose to store renewable energy generated by wind and solar power because batteries still have 60 to 70% of their energy storage capacity (Rücker et al. 2024). Small Li-ion rechargeable batteries (SLBs) provide advantages, such as continuing to create revenue; however, this revenue cannot exceed the cost of the battery, and it also plays a part in reducing production time and cost, as well as preventing the extraction of raw materials, effluent, and CO_2_ emissions (Parviziomran and Elliot 2024); however, it relies on several battery characteristics, including the battery’s age, weight, types, chemistry, and heating value. On the other hand, the direct propagation into the “end life,” disassembly i.e., recycling in a particular way for extracting valuable materials (Parikh et al. 2023) is environmentally questionable process.

Furthermore, there are two crucial economic and environmental factors to take into account when employing these retired batteries: first, if they are acceptable for applications requiring power and energy, and second, how long they will stay to provide financial advantages (Debnath et al., 2014). Less range and more frequent charging are signs of being able to be disposed of, but they do not always indicate the device is meant for trash (Mu et al. 2023). Currently, the creation of strategies for the reuse and recycling of batteries is a major part of EU policies (regulation of the European Parliament and the Council on batteries and waste batteries, repealing directive 2006/66/EC and modifying regulation (EU) No 2019/1020); however, neither the second-life cycle nor the end-of-life processes are fully developed (https://hungarianbatteryday,hu/wpcontent/uploads/Inno-Energy_Reference_Strategy_Final 2021).

Before reusing the EVB pack, “repurposing” and “refurbishing” are possible. Repurposing is “using the pack as it is” (Liu et al. 2023), while refurbishing involves disassembling the packs, remanufacturing the cells, and repackaging them in new modules before using them in stationary applications like factory acceptance tests (Li et al. 2020), telecom towers, micro-energy storage power stations, construction sites, house or a cabin, off-grid residents, warehouses or shed, electric bikes, home energy storage, system, portable mini fan, cool flashlight with 9 V batteries, and backup power for elevator (Höschele et al. 2022). However, numerous problems remain, such as the requirement for extra installation space, the inability to connect packs in series but individually in parallel (Hertel et al. 2024), the system integrator’s lack of warranty on battery performance (life extension, efficiency, charging %), battery manufacturers can only guarantee the estimated residual capacity, and specialist work is required, which reduce cost savings (Guo et al. 2024).

In the end-life process, there is a need to recycle wasted batteries for recovery of precious metals, chemicals, and battery parts after a successful 10 to 15 years using in first life for E-cars and second life for stationary purposes (Fioriti et al. 2023). However, there are mainly two stages: (i) pre-treatment and (ii) treatment process for extracting treasured metal elements, for instance, Li, Co, and Ni, from scrapped batteries (Fallah and Fitzpatrick 2022). Recovering precious metals from abandoned LIBs is essential for reducing supply risk in developed countries. Moreover, long-term solutions must be found to environmental and safety concerns to build an efficient technique for recycling wasted LIBs (Etxandi-Santolaya et al. 2023). “Currently, worldwide, it’s very hard to figure out for what percentage of LIBs are recycled, but the value everyone quotes is about 5% to 7%,” says Dr Anderson (Dai et al. 2020).

However, numerous drawbacks present economically, ecologically, and environmentally for recycling of these used batteries which need to be solved in a useful manner (Chirumalla et al. 2024). This must make sense from an economical as well ecological point of view such as adopting efficient recycling methods as well as environmentally low burden, i.e., water, soil, and air pollution, so there is a need for a right recycling place and/or path (Barbosa et al. 2022). Mainly, firstly, the pre-treatment methods of end-life processes could be mechanical, or thermal, and following such processes, the materials/mechanisms are grouped according to their physical characteristics, for example, particle size, method, mass density, and electric and magnetic properties. The second stage, the extraction of metals such as Li, Co, Mn, Ni from hydrometallurgical, pyrometallurgical electrochemical, and bio-metallurgical or bio-hydrometallurgical methods is a promising recycling method (Kallitsis et al. 2020). Currently, the hydrometallurgical method is more efficient, safer, and eco-friendly than pyrometallurgical and other techniques due to metals’ solubility in acid. While the bio-hydrometallurgical method is a growing technology that draws several researchers and offers potential benefits in recycling an environmentally sensitive method.

In this regard, the objective of this research was to conduct the assessment of currently published articles in the field of first life and second life using recycling methods of wasted E-car LIBs. However, so far, no such kind of cumulative, i.e., first life, second life, end-life using and recycling methods scientometric, social network, and S-curve analysis research was found to be conducted in the field of EVs and LIBs. Therefore, the main goal of this research is to utilize scientometric research to assess, distinguish, classify, identify, and highlight the challenges of extremely impactful research. Notably, the research identified the technological development processes, hotspots, and trends by using an S-curve derived from an article database, which can provide quantitative and qualitative scientific guidance for future EV battery recycling research. The contributions of the research are as follows: providing detailed information and analysis of highly published articles by countries, journals, and authors on second life and recycling design method of wasted LIBs, highlighting the existing research gaps, issues, and challenges to develop as economically and environmentally efficient second life and recycling technologies. Moreover, based on the current research discussion and review, a few future research suggestions and ideas are provided.

## Methodology

**Fig. 1:**
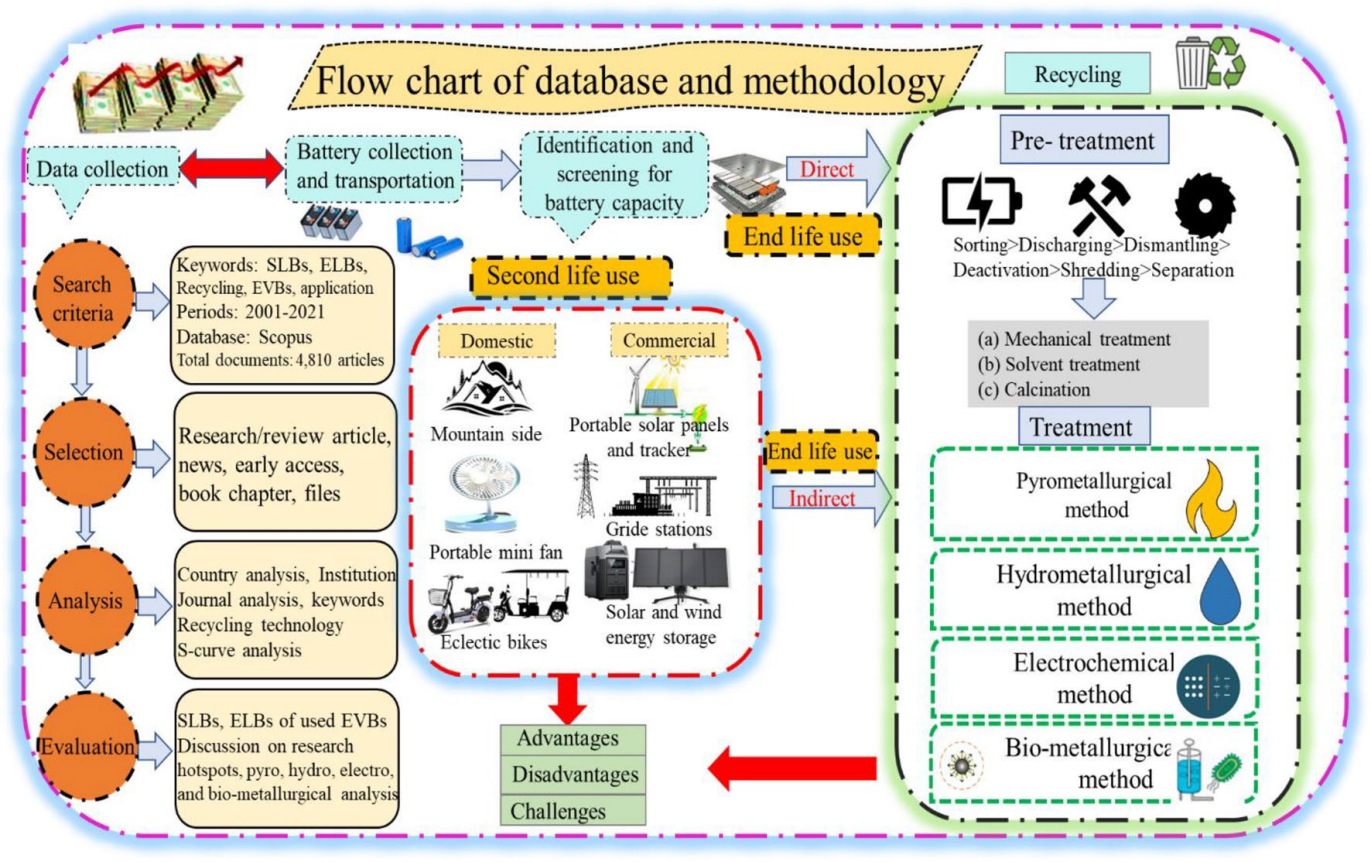
The methodology of second life usage and recycling design of wasted Li-ion EVBs

This research is evaluated using a Scientometric technique, which is a widely known scientific methodology. A scientometric analysis is described as a statistical technique used to examine research publications with an emphasis on citation. Hence, this research seeks to identify the most publications on EVBs and evaluate second life, end life, and recycling methods. The data evaluated in this paper was chosen through three processes to identify the present state of academic understanding with relation to the recycling of lithium from wasted LIBs: (i) authors using search criteria to find published papers in Scopus databases; (ii) besides using a list of questions to extract critical information and research gap from the chosen literature; and (iii) classifying the literature according to the variations in the main separation phases used in recycling systems. The overall methodology of this research is shown in Fig. 1. This research prioritizes research articles over patents and other sources for bibliometric analysis because they better reflect scholarly impact. Research articles are more accessible, align closely with the research’s scope, and typically contain citations that indicate academic influence. While patents offer insights, they may not always be relevant to the analysis’s specific question or scope. Therefore, focusing on research articles maintains a clear and coherent research focus (Cabeza et al. 2020).

### Selection standards for relevant literature

Research used the following criteria and limitations to search for literature in Scopus and Google Scholar between January 2001 and December 2021: (“used batteries”, “LIBs or lithium-ion batteries” “recycling of EV batteries” OR “end-life of batteries” OR “treatment technologies” AND “recycling methodology”. Research articles and review articles have been selected as the filter options for article type. The publications found from the initial search were then manually filtered by reading the abstracts.

Studies that (i) did not concentrate on the causes and methods of precious metal extraction, (ii) did not take lithium recovery into account, and (iii) only looked at reuse and regeneration were eliminated (recovery and recycling were not studied). The literature was expanded further by reading the references of the papers found when analyzing other research to incorporate as much relevant information as feasible.

### Objective to extract and solve the information

- What you mean by second life using and end life or recycling?
- What is the history and contribution of countries, organization, and journals in second and recycling of used batteries?
- What role have governments, organizations, and journals played in second life and recycling?
- What are the key sources for second use of EVBs?
- What are the challenges in second-life stage using of batteries?
- How difficult is EVB recycling technologically and economically?
- What innovative technologies are working for second-life using and recycling of used batteries?
- How wasted LIBs: a chore or a chance for new business?
- Which recycling technologies are economically and environmentally beneficial?
- What will be the future of wasted Li-ion EVB’s second-life using and recycling technologies?

### Social network analysis

Social network analysis (SNA) is a measurable tool that may be used to analyze and visualize the theoretical collaboration of research among countries, universities, scholars, and journals. Due to its ability to continually monitor interactions, build system concepts, and control processors, SNA is commonly used to assess the impact of groups and take a strategic approach to teamwork (Zahoor et al. 2022).

In this research, the data from scientometric analysis can be used to visualize the network among countries, authors, journals, institutions, and constructive keywords network to understand the better results of collaboration patterns (Shiikha et al. 2023). Evaluating these networks using VOSviewer software for data mining from the raw data and then using pajek software better visualize the network relationship (Zahoor et al. 2023a). We can understand researcher interactions and, more critically, research information flow as well as improve knowledge activation and research communication skills.

### S-curve analysis

Several simulation techniques for predicting the future of technology have been explored. Since it is predicted that a product or technology would follow an S-curve, several researchers have utilized the S-curve to model the evolution of technology to determine its level of maturity. In this research, S-curve analysis was used to identify the present situation and future development of treatment technologies (Zahoor et al. 2023b). A logistic model is used to quantitatively analyze the publication related to treatment technologies and build S-curve graphs to show the results (used Log-let Lab 4 software developed by Rockefeller University), Eq. 1 depicted

1 $${Y}_{t}=\frac{K}{1+{e}^{-a\left(t-b\right)}}$$

where *Y*_t_ is annual cumulative publications that denote the dependent variable, *t* is the time variable for the S-curve, *a* and *b* are model parameters, and *K* is the value of maximum published articles in this research.

## Results and discussion

It is difficult to identify and understand the current research trends as well as the most distinguished research in a specific area to identify and expand that field. The research objectives are to (1) assess the relevance of current research trends and (2) locate relevant publications in the fields of second life, end life, and treatment methods.

### Distribution source and annul publication

**Fig. 2:**
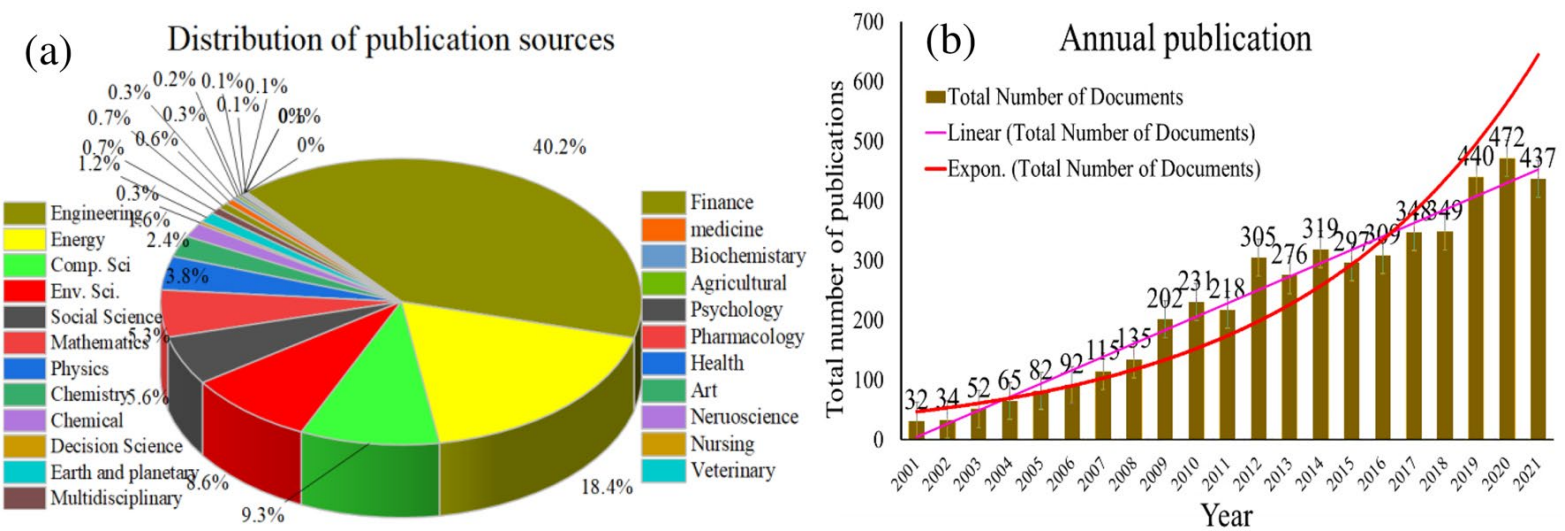
**a** Percentage distribution of publication sources, and **b** the annual distribution of publication of research articles in wasted Li-ion EVBs from 2001 to 2021

Analyzing the distribution sources and annual publication trends is valuable for understanding the landscape of research within a specific field, assessing publication patterns, identifying research trends, evaluating research impact, and informing decision-making processes at various levels of the academic community. Figure 2 a shows the relative contribution of articles, reviews, meetings, abstracts, proceeding papers, and other types of documents formed. The highest number of publications was found in different fields such as engineering about 40.2%, energy at 18.4%, computer science at 9.3%, environmental science 8.6%, and social science 5.6%, while the least publications about 0.1% in nanoscience, nursing, and veterinary fields.

In the Scopus database, a total of 4810 research articles were collected via the use of manual screening and proofreading from the years 2001 to 2021. Figure 2 b shows the annual publication of wasted Li-ion EVBs from 2001 to 2021. According to the investigation from 2001 to 2011, the number of publications increased steadily, and both linear and exponential trend lines depict the ascent in ascending order of the number of publications, while from 2012 to 2018 was the quite similar number of publications. The number of publications has gradually increased, reaching 472 articles in 2020, and 437 articles were registered in 2021. This trend can be defined in part by the point that with the development of the industrial revolution and increasingly serious problems of wasted batteries, the number of articles related to second life usage and treatment methods increased from 2018 to 2021.

### Geographical distribution analysis

Analyzing the geographical distribution of research publications on EVs and power batteries serves several purposes: identifying research hotspots, understanding regional or country priorities, tracking technology innovations and transfer, policy formulation and regulation, and market insights (Hung et al. 2021). This section indicates that the authors utilized specific software tools VOSviewer and GIS, to analyze the collected data and get a picture of the top research publication countries from 2001 to 2021. This software facilitated the systematic analysis of bibliographic data to gain insights into the global research landscape surrounding technologies related to EVs and power potteries (Hu et al. 2020).

**Fig. 3:**
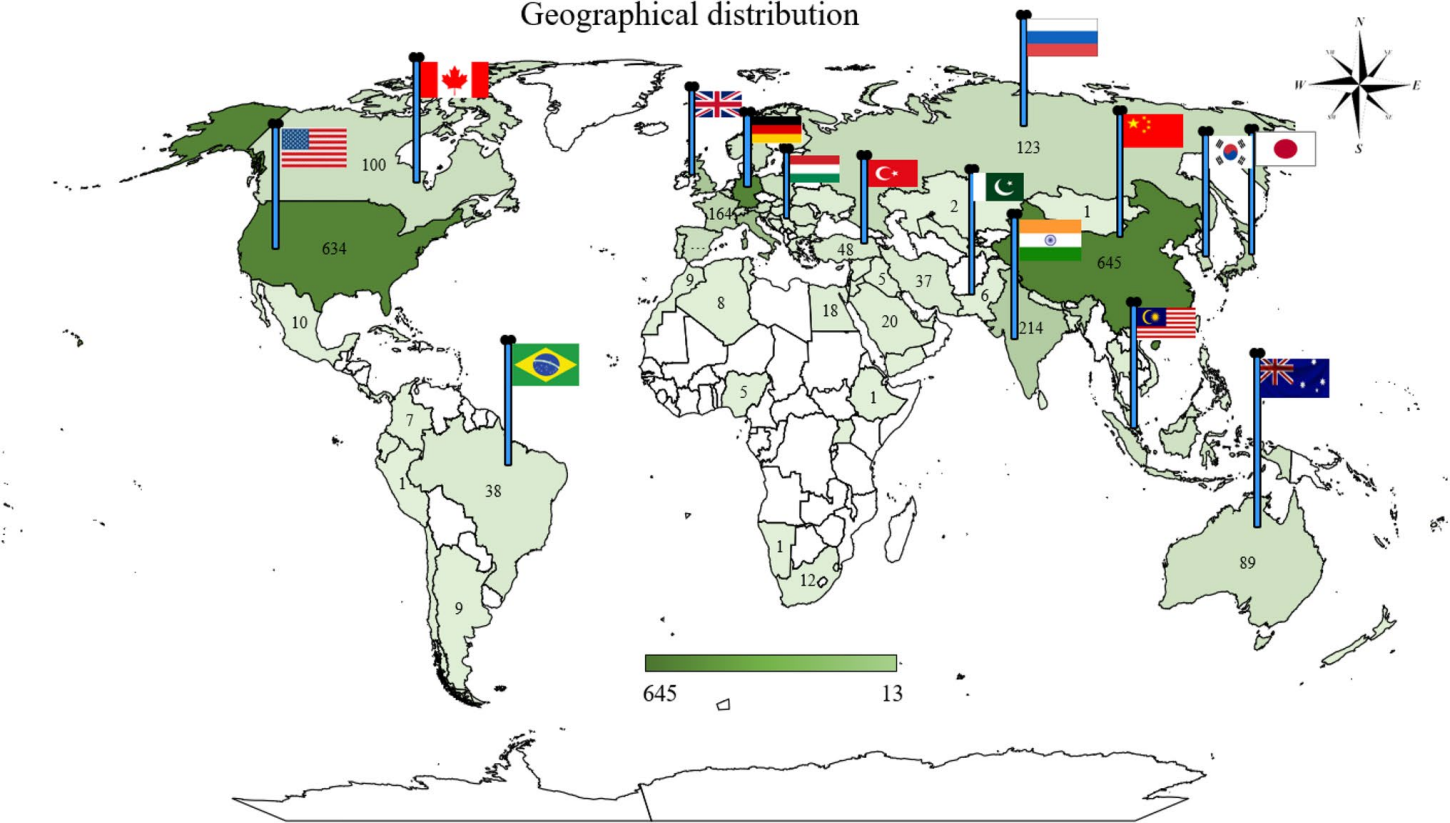
Geographical distributions of publication from 2001 to 2021, the dark green to light green color show different numbers of publications of countries

The findings presented in Fig. 3 reveal that several countries stand out as leading contributors to scholarly publications within the field under examination. Specifically, China leads with 645 publications, followed closely by Germany with 635 publications, the USA with 634 publications, Italy with 306 publications, and the UK with 249 publications, as of the year 2001–2021. Conversely, India, Japan, France, Russia, the Netherlands, South Korea, Spain, Sweden, and Canada have demonstrated relatively lower levels of scholarly output, each accounting for publication counts ranging from under 214 to 100 during the same period. So, China, Germany, the USA, Italy, and UK have some of the most advanced technologies for recycling or second-life usage of EVBs, which has allowed them to lead the way in producing research and publications about the topic (Zhou et al. 2020), (Hoyer et al. 2015). Moreover, these countries are known for having top-level universities and research institutes that are devoted to technological advancement and research, so it makes sense that they would be the ones leading the way in the second-life usage and recycling of LIBs. Besides, they also have high levels of funding available for research and development which has allowed them to make great steps in this field.

That’s why most recycling firms are placed in China with the help of manufacturing partner companies (Cabral-Neto et al. 2022). According to the Ph.D. scholar (power battery and materials analyst) Shalu Agarwal from Yole, “Most automakers are currently comparing various recycling businesses and looking for the finest battery recycling partners for their companies.” Moreover, the USA and some European countries such as Poland, Switzerland, Germany, France, Norway, and the UK established recycling plants as well. For instance, redwood Materials led by Tesla co-founder J.B. Straubel said it will build its battery materials manufacturing (100 GWh per year of cathode active materials) and recycling facility in the USA for 1 million EVs by 2025. Overall, geographical distribution analysis of research publications efforts can provide valuable insights for stakeholders across academia, industry, and government, helping to guide further strategic decision-making, collaboration efforts, and policy development in this rapidly evolving field.

### Most productive journal, author, and institution analysis

The journal, authors, and institution analysis graphs are made by VOSviewer software to check the most successful publications, citations, and collaborations on second life usage and end life or treatment methods of wasted EVBs. The different colors and group clusters show the different research collaborations’ interests. Total citations and total link strength (TLS) are the two basic weight attributes to analyze and represent the quality and quantity of given published papers. Moreover, the importance of journals, institutions, and researchers’ performance is judged by the number of publications and citations.

**Fig. 4:**
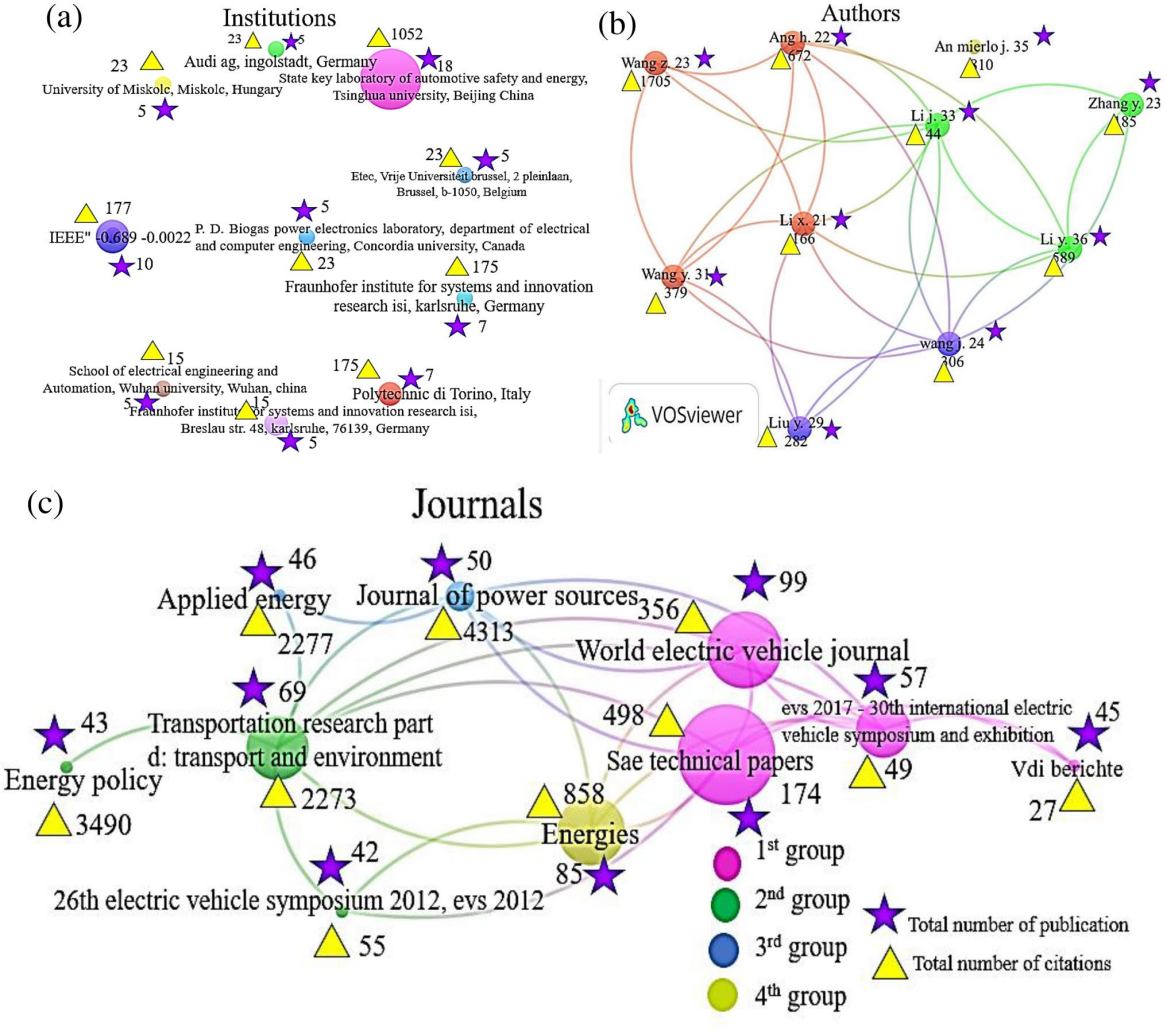
**a** Institution’s performance, **b** the author’s publications and collaborations, and **c** the most influential journals. The different colors point out the cluster to which a journal had high publications, and the strength of the lines describes the collaboration and teamwork with other institutions, authors, and journals given by the clustering

Institution or organization analysis illustrates the interest in collaborations of research, sharing ideas and introducing innovations, and helps to enhance the research methodologies. For institution performance in Fig. 4a, we select the criteria for co-authorship of organization or institution as follows: minimum number of publications of five and at least four citations collectively; we extracted the ten top institutions which meet this criterion. Unfortunately, there is no collaboration among these institutions, and they might be with other institutions in the whole data or error of the software.

A higher number of publications and citations of institutions show higher research interest over other institutions. The first position with 18 publications and with 1052 citations in China is “State Key Laboratory of Automotive Safety and Energy, Tsinghua University”. Secondly “IEEE” comes second with 10 publications and with 177 citations. Third are “Fraunhofer Institute for Systems and Innovation Research, Fraunhofer ISI, Karlsruhe”, Germany, and “Polytechnic di Torino,” Italy, with equal seven publications and different citations 175 and 27, respectively. The other remaining institutions have published the same five articles and gained less than 30 citations until now.

In Fig. 4b, there are different four color groups: brown, purple, green, and yellow which show the author’s collaborations and cited the articles by other authors. The start describes the number of published documents, and the yellow triangle describes the total number of citations gained by whole publications. The Chinese author Li Y has the highest number of 36 publications, and his papers are cited 589 times by other authors as well as higher TLS. The second author whose name is An Mierlo J. has 35 publications and 80 citations, but there is no TLS, i.e., no author who collaborated and cited his papers and his papers might be cited by other authors in our research data or other researchers, that’s why he is alone in his group. Third is Li J with 33 publications and 44 citations; he scored very least citations which may be due to the low quality of his research or not wide assessment of his research for other researchers. Moreover, Wang Z has the highest citation of 1707 on 23 publications and higher TLS which shows that Wang Z has innovative and good research papers. Hence, we can assess other remaining authors’ role and performance in the field of recycling technologies of used LIBs. The author’s analysis concluded the similar research interests, help and communication channels among other authors, the importance of research effort, and recognition mechanism for researchers and institutions. In conclusion of this section, all journals, institutions, and authors’ publications show the importance of research on second life and treatment methodologies of wasted Li-ion EVBs.

The selection of journal analysis in this research may not have focused only on the most prominent journals; the authors aimed to include a diverse range of sources to provide a comprehensive analysis of the research issues. So, the selection process was based on relevance to the research question and the availability of reliable data. Consequentially, in Fig. 4c, the total of 174 articles, 498 citations, and 6 TLS, the journal *SAE technical papers* is the one with the greatest journal for second life usage and end life or recycling methodologies of wasted EVBs. It is demonstrated to be more influential than other journals in this field. The second journal, *World electric vehicle* journal, has a total of 85 articles 858 citations, and 52 TLS. The third is the *Energies* journal with higher citations and TLS which demonstrates that they are reliable journals. Additionally, the performance of other journals can be assessed in the graph.

### Keyword co-occurrence analysis

**Fig. 5:**
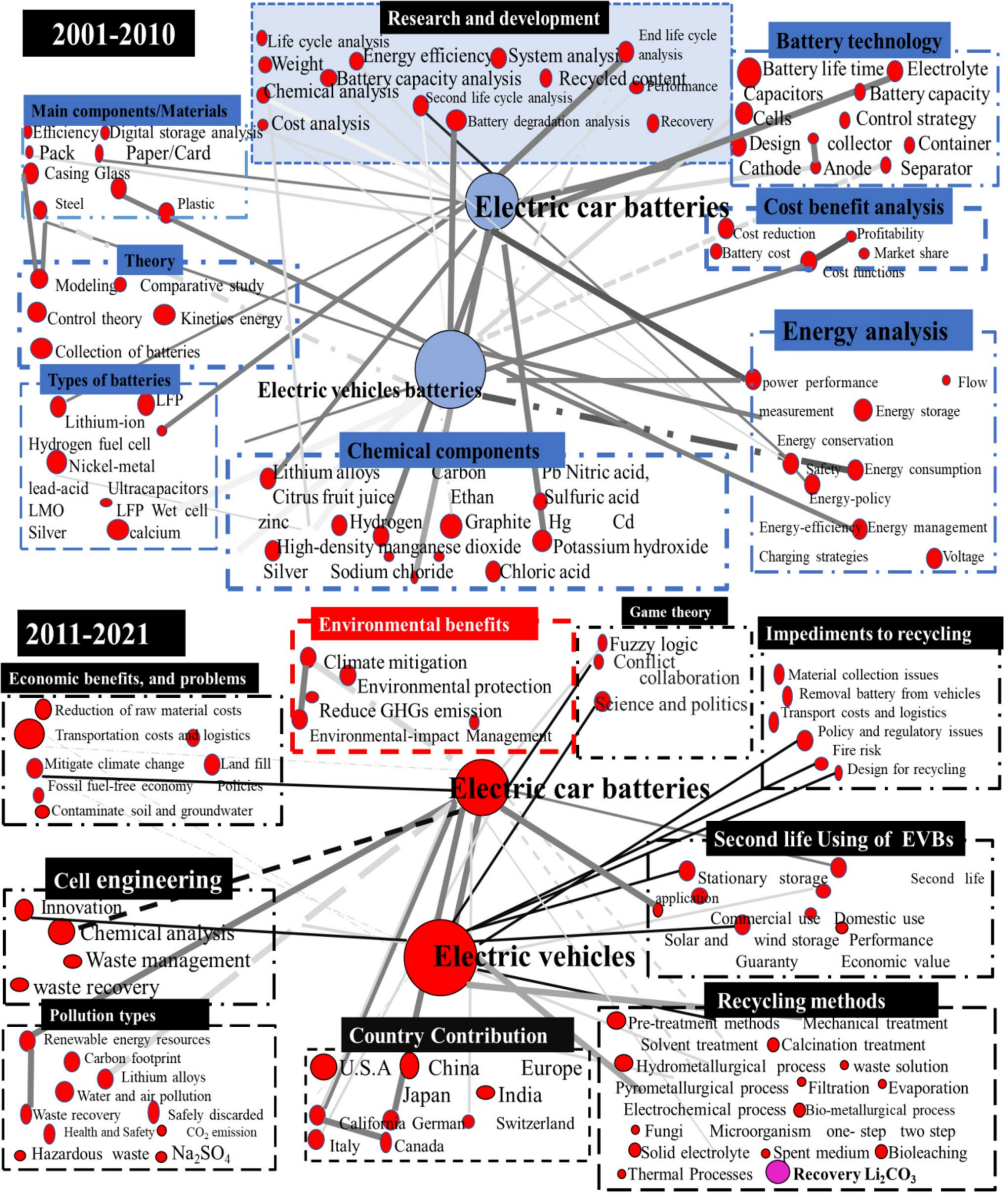
Network of connections and keyword evolution in publications between 2001 and 2021. The size of the red dots indicates the proportional quantity of keywords. The red dot’s size indicates how frequently a particular keyword appears or was used in the linked articles. The grey line pattern represents the number of co-occurrences. The greater the thickness and darkness of the line, the greater the frequency of co-occurrences of the two terms used by the writers

It is vital to go deep into each document and identify the key terms to confirm the subject matter and core themes of research on second life usage and treatment methodology of wasted LIBs. This study is crucial for finding patterns in developing topics and hotspots that might be relevant for areas of research, development, and innovation in the domain of wasted EVBs. Out of a total of 4810 articles, 28,860 results were produced by the analysis of keywords connected to wasted EVBs. Furthermore, we extracted the most famous and close to our research keywords about 178 to 200 words which reached the limits of focus on research direction. Among them, we divided our data into main two parts, 2001 to 2010 and 2011 to 2021, and then this data was further classified on behalf of their terms and conditions for understanding the research trend, progress, development, and research gap which can be seen in Fig. 5. Main research information data or term types from 2001 to 2010 of EVBs: “battery technology,” “theory,” “cost and benefits,” “energy analysis,” and “chemical component analysis” mostly have been used.

From 2001 to 2010, researchers, companies, institutions, and governments focused on the research and development of EVBs, the early stage of improving and developing distinct EVB pieces. R&D encompasses basic, applied, and development research. Basic research uses theory, modeling, comparative study, and control schemes to get new information and technologies to develop and innovate the research area without any specific application or usage of experimental instruments; some examples are energy storage capacity analysis, battery design, weight analysis, LCA, and end-life assessment. Applied research discusses scientific study and research that seeks to solve and find practical solutions to problems. Development research is a field of research aimed at discovering, investigating, and testing new methods, processes, or approaches that promote development. Moreover, development research often leads to the invention of new products and processes or the improvement of existing ones.

Another classification of research direction from 2001 to 2010 is battery technology which included some important keywords for instant, battery lifetime, and battery capacity which depend on the battery components such as cell, electrolyte, anode, and separator. Furthermore, battery technology and performance are also directly and indirectly interconnected to the origin and type of the chemical components such as Li, C, Ni, Hg, Zn, H^+^, graphite, high-density manganese oxide in the process of the analysis of the LIBs for battery, storage, performance, and lifetime of the battery. The main classifications of research direction from 2001 to 2010 were introducing new batteries such as LFP, NCM, NCA, and hydrogen fuel cell, for high storage and safe usage in EVs. For these purposes, the research mainly focused on the analysis of battery chemical components and energy analysis. The main factor during the period of 2010 to 2011 was cost benefits analysis from battery production, selling, and approach to buy for consumers, because some batteries are very expensive due to expensive chemical material and high taxes by the government. To reduce the price and environmental importance of new batteries and vehicles, researchers provide some game and fuzzy theories.

From 2011 through 2021, the predominant trend in research shifted from basic and applied research to development research for environmental friendliness, better performance, and long-life lifetime. The core research keywords in this section which mainly focus on development research are the following: “components and material,” “size and cell engineering,” energy storage,” “second life use,” “stationary use,” “recycling technologies,” “impediments of recycling,” “environmental benefits,” “pollution control,” “role of countries contribution,” and “economic benefits.” It has been seen that in the last few years, scholars and researchers have paid more attention to R&D in pollution-free, environmentally friendly production; raw material and money saving; better performance; and second life and recycling methodologies. Recycling saves resources, promotes the economy, and creates employment (Canals Casals et al. 2019). Sustainable material management emphasizes the productive and sustainable use of resources throughout their entire life cycle while minimizing environmental effects and preventing waste and increased exploitation of natural raw materials.

Besides economic and environmental friendliness, there are some impediments during the recycling of LIBs, for instance, removal of batteries from vehicles, material collection issues, transport cost and logistics, recycling policy and regulatory issues, fire risk during transfer and disassembly, and design for recycling. During transportation, some issues of transfer rules and policy regulations such as permission from one place to another place, risks of overloading, battery leakage, incomplete wrappings, and toxic chemical leaking cause fire. The transportation problem can be solved by providing soft policies and rules and adopting safety precautions. A cost–benefit analysis compares the expected or estimated costs and benefits (or opportunities) connected with a project choice to evaluate if it makes business sense. Cost–benefit of new production, second life use, and recycling used EVBs help to calculate the gap between economic and environmental losses. Many developed countries such as the USA, China, and European countries: Germany, France, Italy, Denmark, Hungary, Switzerland, and Poland are taking interest and good incentive for the recycling of waste LIBs. Moreover, these countries are providing basic facilities, convenience, policy and regulation issues, and opportunities for recycling companies and organizations.

In the keywords section, there are some valuable and convenient recycling technologies proposed such as pyrometallurgical, hydrometallurgical, bio-hydro metallurgical, and electrochemical which are being used by recycling companies, organizations, and researchers. These recycling technologies have different comically and environmental advantages and disadvantages. For the recovery of metals from used batteries, the used of citrus fruit juice and fruit peels is environmentally and comically favorable (Burggräf et al. 2020). In the recycling procedure of batteries, the carbon, harmful gases, heat, water and air, health safety, respiratory, and production hazardous waste are generated which are harmful to the environment. The keywords co-occurrence analysis concluded the whole research on EVBs and how to move from basic research to development research during the last 20 years. Researchers and scholars pay attention to the improvement of battery’s internal structure (anode, cathode, electrolyte, etc.), external structure (size, body cover), storage capacity, first lifetime use age, environmental and economic benefits, second lifetime to end life time, recycling technologies, and recovery of metals.

### Second life of used EVBs

**Fig. 6:**
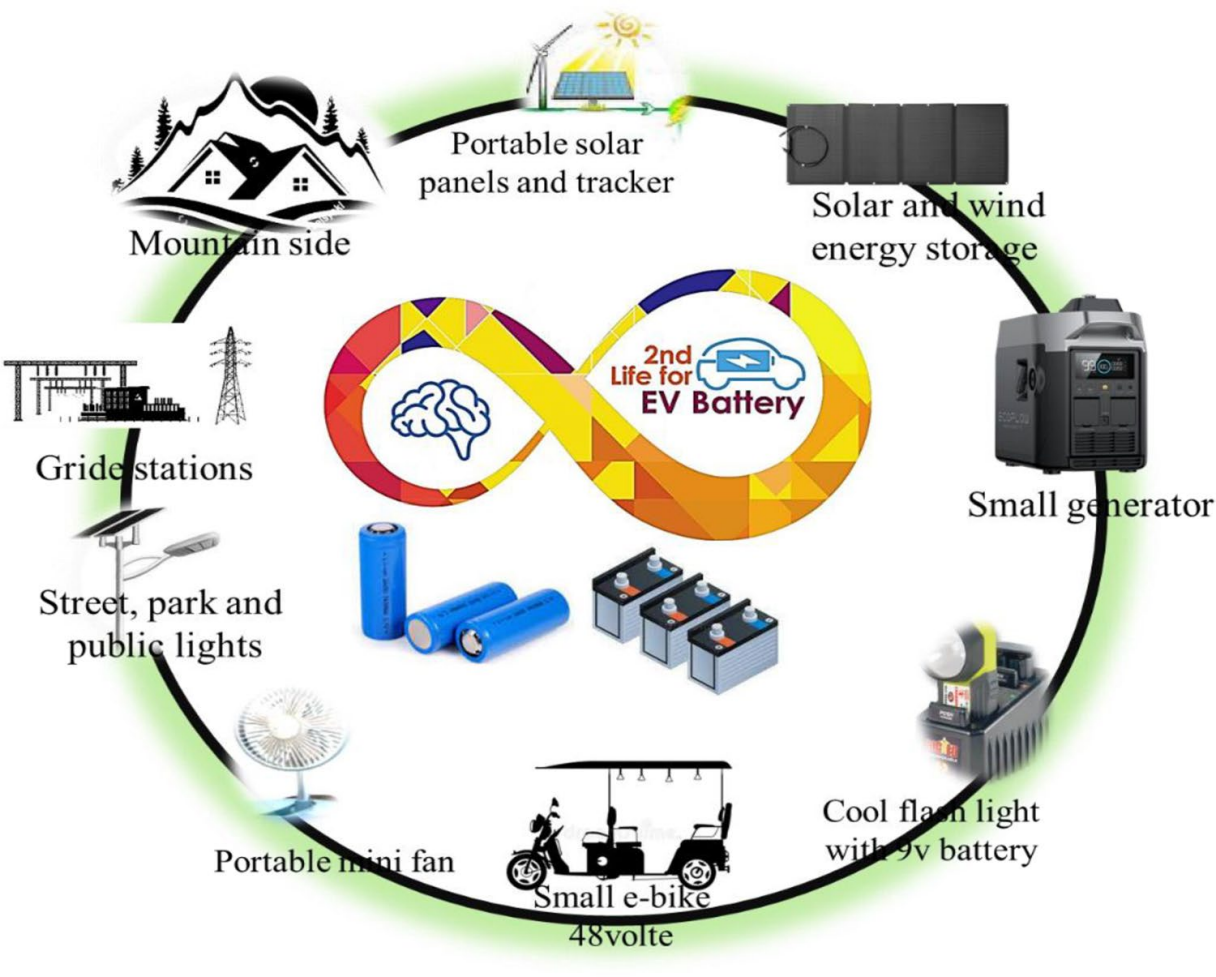
Domestic and commercial use of SLBs in different sectors

Second life batteries (SBLs) are used for domestic and commercial energy storage purposes for short-term peak power, minimizing the chance of power outages and secondary services (Chirumalla et al. 2023). Some of the applications are mentioned in Fig. 6. For the storage of solar, wind, and tidal energy, an energy storage system can be used where there is no direct access to electricity from the grid station such as top of the mountains and long-distance houses and cabins (Casals et al. 2019). Moreover, used batteries could be useful in operation of protective devices, emergency lighting at generating stations, e-bikes and rickshaws, for starting ignition and lighting automobiles, aircraft, steam engines, and diesel railway trains (Al-Alawi et al. 2022), as well as small cars and trains which run in the picnic parks. It might be dangerous to repurpose EVBs at home. The battery packs are large and can explode if they are damaged.

Second-life batteries from EVs have gained attention as a promising solution for repurposing batteries after their initial use in vehicles. There are few real-world case studies demonstrating the utilization of second-life EV batteries. In China, where EV adoption is significant, companies like BYD company have been actively exploring second-life applications for EV batteries. One notable example is the partnership between BYD and Shanghai Electric Group to deploy second-life EV batteries in energy storage projects (Tao et al. 2022). These batteries are used to store renewable energy generated from sources like wind and solar for later use, helping stabilize the grid and support sustainable energy initiatives. In the USA, companies like Tesla and GM are also investing in second-life battery projects. Tesla, for instance, has deployed Powerpacks and Powerwalls utilizing repurposed EV batteries. Additionally, GM has partnered with companies like ABB to explore the use of second-life EV batteries in grid applications. These batteries can help improve the reliability and efficiency of the electrical grid while supporting renewable energy integration (Schulz et al. 2020).

On the other side, in the Netherlands, the Dutch startup Alfen has been involved in repurposing EV batteries for grid-scale energy storage. In collaboration with Renault and The Mobility House, they have implemented a project called “JUMP,” which utilizes second-life EV batteries for grid stabilization and peak shaving. These batteries are integrated into Alfen’s energy storage systems, providing grid services like frequency regulation and peak load management (Quan et al. 2022). In Japan, Nissan, a pioneer in the EV industry, has been exploring various applications for second-life EV batteries. Through this initiative, Nissan aims to refurbish and repurpose EV batteries for stationary energy storage systems, such as those used in homes and businesses. These systems help store excess renewable energy and provide backup power during outages (Moazzam et al. 2021). These case studies illustrate the diverse applications of second-life EV batteries, ranging from grid-scale energy storage to residential and commercial use. By repurposing EV batteries, stakeholders can extend their lifespan, reduce waste, and contribute to the development of a more sustainable energy ecosystem.

Some economic benefits of application of SLBs prevent the mining of natural resources, reinforce country economy, and produce jobs. Apart from the economic perspective, the use of SLBs will also have an impact in an environmental viewpoint (Koroma et al. 2022). The concept of zero waste management is applied by using SLBs to prevent the creation of waste as well as reduce the demand for environmentally harmful material extraction which needs new batteries, water for mining, CO_2_ emission, and electricity for cell manufacturing. The cost of a second reused battery is approximately $50/kWh, compared to $200–300 for new battery construction today and could stay competitive at least until 2025 to 2030, when the price of a new battery is expected to reach $90/kWh (Gupta et al. 2024).

1.1.1.1.1. Advantages and disadvantages of second-life batteries

Some advantages of the application of used batteries are half of the price of new batteries, new companies established for reconditioning discarded batteries resulting in new jobs opportunities, increases in the growth of the recycling companies, circular economy and zero waste concept, and less demand for new batteries resulting in less mining of raw material, mining, and CO_2_ emission (Dai et al. 2019).

2.2.2.2.2. Challenges of second-life batteries

Notwithstanding the potential advantages of the SLBs, the implementation needs to overcome some obstacles and challenges: Regarding implementation, standards and automatization will speed up their industrialization, having a positive contribution in terms of safety and cost. As explained earlier, the dismantling process will require highly qualified professionals who can have an impact on the cost–benefit of SLBs. In addition, the battery comes in different shapes and forms together with different voltages and chemistries. This is a real challenge for the reconditioning process and might require additional assessment; thus, it can further increase the reconditioning cost.

The finding a similar cell and matching the good battery together is an important factor for the performance and lifespan of SLBs (Wang et al. 2022). The commitment is that the full implementation of the SLBs impacts the price of new Li-ion batteries and their cost–benefit balance positively. Moreover, the critical challenge is to extend the life cycle of these SLBs by more than 5–10 years in response to what the customer may have in mind compared to new batteries (Tolomeo et al. 2020).

### Recycling methodologies of wasted Li-ion EVBs

Treatment methodologies are a major component of any research paper dealing with economic, social, and environmental problems, as they offer potential solutions for addressing the issues presented. By including a section on treatment technologies in a research paper, the writer can provide a comprehensive summary of available options for dealing with a particular comical and environmental concern. Additionally, the writer can evaluate the effectiveness of the available treatment technologies and suggest the best course of action for future research (Liu et al. 2019b).

The separation of the battery from the vehicle is called disassembly which is the most important and critical step because it is very heavy and connected to the vehicle with electric wires. In the next stage, dismantling and recycling processes start (Yun et al. 2018). Four different processes are as follows: pyrometallurgy, hydrometallurgy, electrochemical, and biometallurgy or bio-hydrometallurgy methods.

#### Pre-treatment methods of wasted Li-ion battery

In the pre-treatment process, firstly, the whole electric wires must be unplugged and disconnected. Secondly, separate the pack by using a chain pulley and put in the yard for further separation. There are some problems with disassembly of batteries as follows: high voltage and wiring, different sizes and shapes of batteries and EVs, pack formations and settings, fixings and tooling essentials, head of bolts, and the position is not always the same. Cells trapped together in modules with gum chemistries not always identified, Deficiency of labeling and identifying symbols related to battery conditions and life (Windisch-Kern et al. 2022).

Some precautions and skills are needed to take things apart, like high-voltage training and safe tools to keep the battery pack from short-circuiting. The qualified employees are required for such dismantling of weight and high voltage of the electric battery. This is a difficulty for an evolving sector with a skills gap. In addition, a computerized robotic system can be used for the disassembly of battery packs for removing the risk of human employees, as well as reducing the cost. This system is economically feasible for automotive industries (Thompson et al. 2020).

**Fig. 7:**
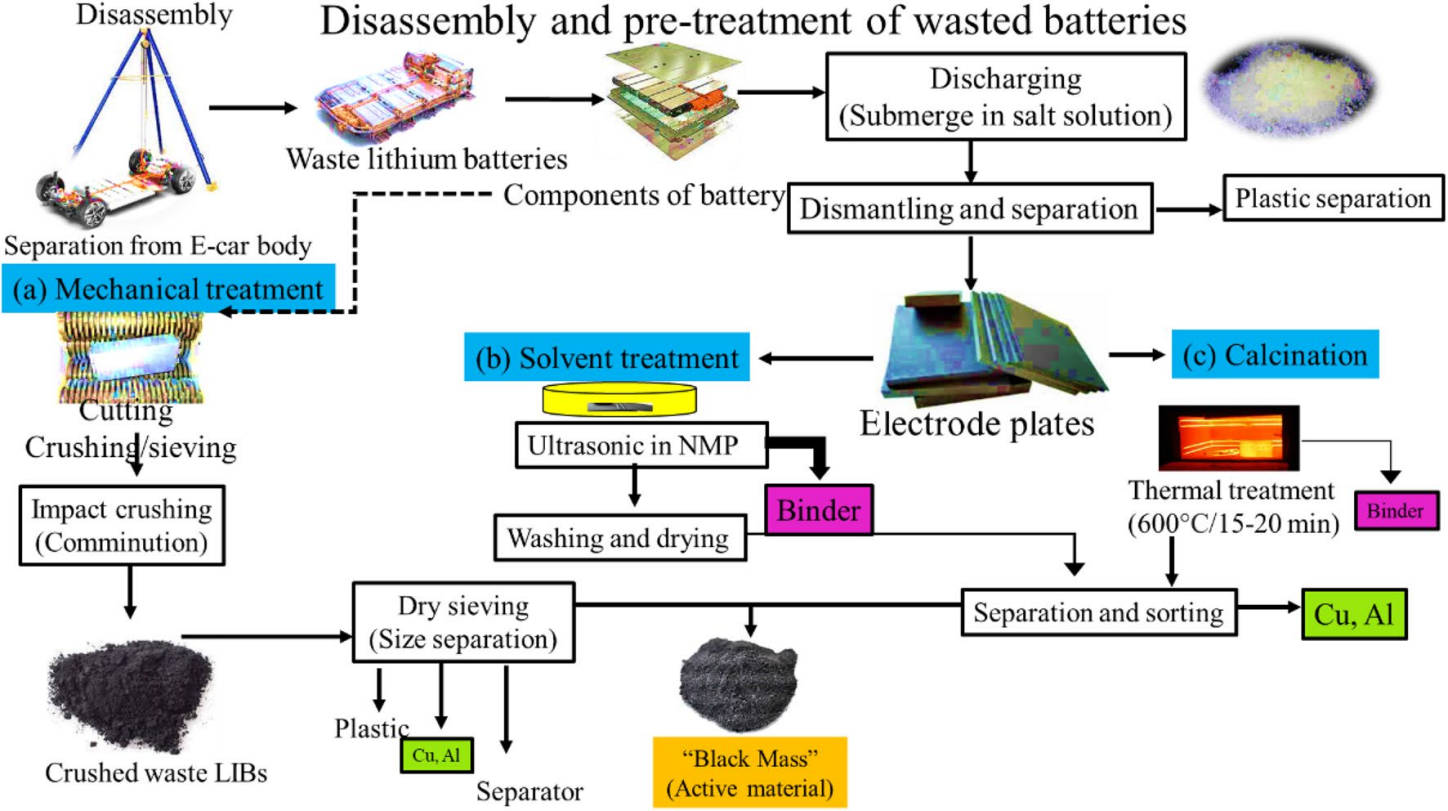
Mechanical, solvent, and calcination pre-treatment technologies and procedures for waste LIBs

After dismantling of battery from the EVs, there are mainly three processes: (a) mechanical treatment, (b) solvent treatment, and (c) calcination treatment for extraction of a key intermediate product, here is so-called black mass. This black mass is a product of used batteries, and this yield is black mass. These three different processes and their steps can be seen in Fig. 7. These three techniques have different economic and environmental benefits. Black mass conation is Co 6%, Mn 6%, Ni 20%, and Li 3.5%, but also carbon and many contaminants such as plastic. Typical black mass composition is Co 6%, Mn 6%, Ni 20%, and Li 3.5%, F 3%, P 0.5%, Cu 2%, Al 2%, Fe 1%, Zn 0.1%, Ca 0.1% beside the 40 % of the main Carbon content (Roy et al. 2021b). Chemical treatment is necessary to remove the valuable metals from black mass. This black mass contains the active material of the battery. They also include carbon or graphite from the anode and the valuable material from the cathode (Selmi et al. 2022).

Here, we put particular focus on Li, Ni, and Co metals. As we can see, there are a lot of other products. We have a large number of contaminations with other metals, and this is due to the fact that the sorting step is not overly precise or clean. In addition, we have elements such as fluorine which is used in the binder that is needed to assemble the battery, and it is also the part of electrolyte. You can imagine we cannot take that material and turn it into the new battery; instead, the black mass will have to be processed chemically in such a way so as to enable meaningful full separation and recycling of individual materials.

#### Hydrometallurgical and pyrometallurgical methods

1.1.1.1.1. Hydrometallurgy process: high recovery rate and Co, Ni, Cu, and Li are recycled. Moreover, manganese and graphite were recycled at moderate temperatures, low energy requirements, and no toxic waste by-product. However, expensive initial investment, rigid procedures, and extensive byproduct waste are needed to settle down. Both approaches need a rise in lithium output, either in terms of product or cost savings (Liu et al. 2019a).

**Fig. 8:**
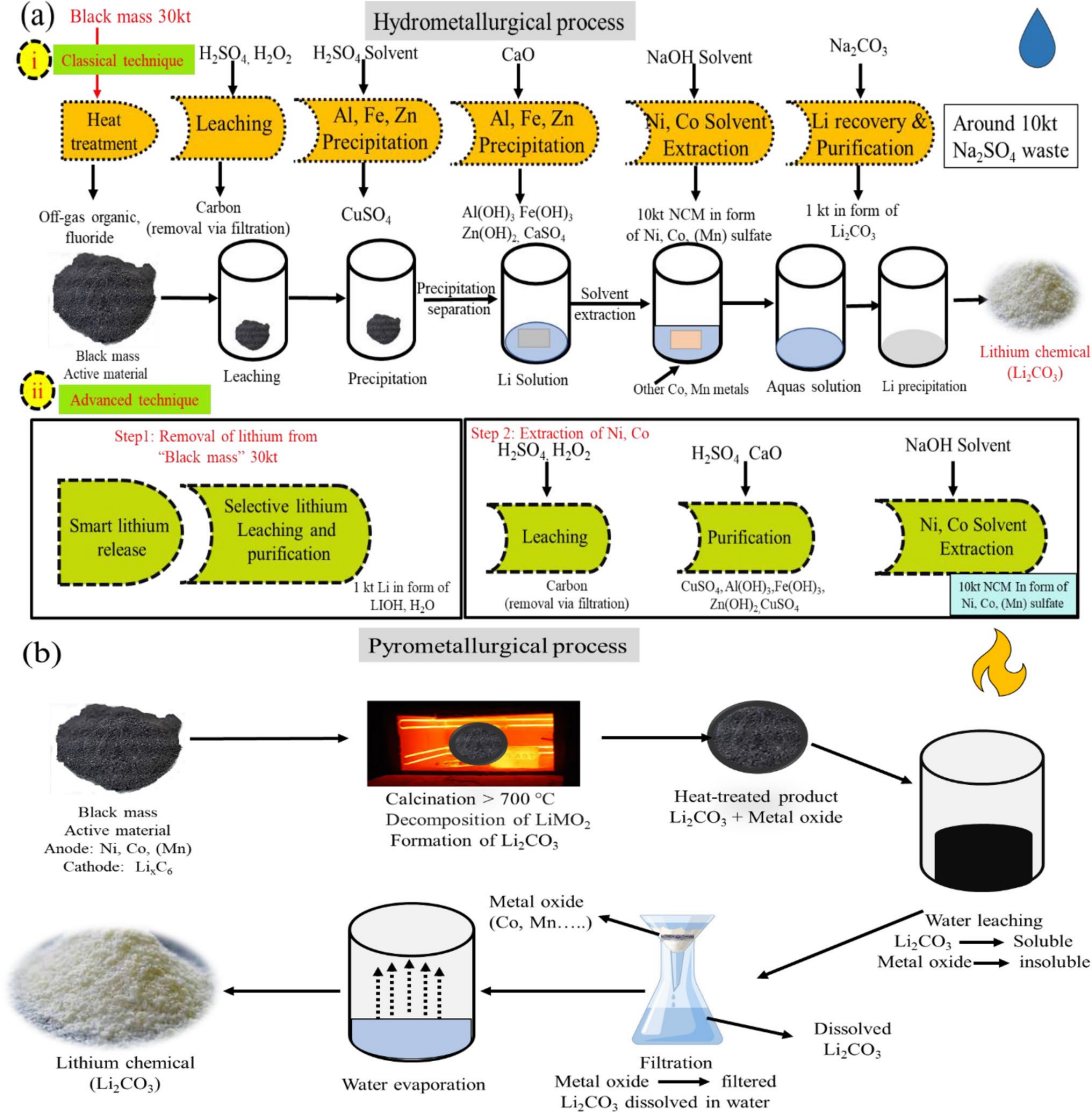
**a** Descriptive process of deep joint hydrometallurgical processes of wasted Li-ion EVBs. (i) The classical hydrometallurgical technique of Li_2_CO_3_ extraction, (ii) the advanced hydrometallurgical technique of extraction of LiOH from black mass. **b** General process of recovering lithium by pyrometallurgy technique

In Fig. 8, step (i) can be seen, and in the first step, heat is applied on black mass to destroy the organic components, while in the second step, metals are leached with the help of H_2_SO_4_·H_2_O_2_ and as a result gets sulfuric (H_2_SO_4_) solution containing all those metals that can be further processed for extraction of Li metal (Brückner et al. 2020). Throughout the end of the chain, we picked out the cathodic materials Co, Ni, and finally Li_2_CO_3_ and Na_2_SO_4_ as by-product at the very end. Each ton of Li produces 10 tons of Li_2_CO_3_. This is not very efficient, but if you use this process to produce cathodic material, all is ready to lower the carbon footprint by about 25%, so this is good for researchers, the environment, and customers in the automotive industry because they keep looking for waste improvement via the carbon footprint. Likewise, this is how Co and Ni from the mine are treated and purified as well (Jiang et al. 2017), besides, there are a lot of technologies around the world with reliable testing and trial process for the extraction of precious metals (Wang et al. 2018). However, currently, modern battery material does not want to use Li_2_CO_3_, and companies want lithium hydroxide (LiOH) to make modern EVBs. The lithium carbonate can be converted into LiOH, but this means yet an additional step and investment. It means additional chemical required that mean carbon footprint increases.

**Fig. 9:**
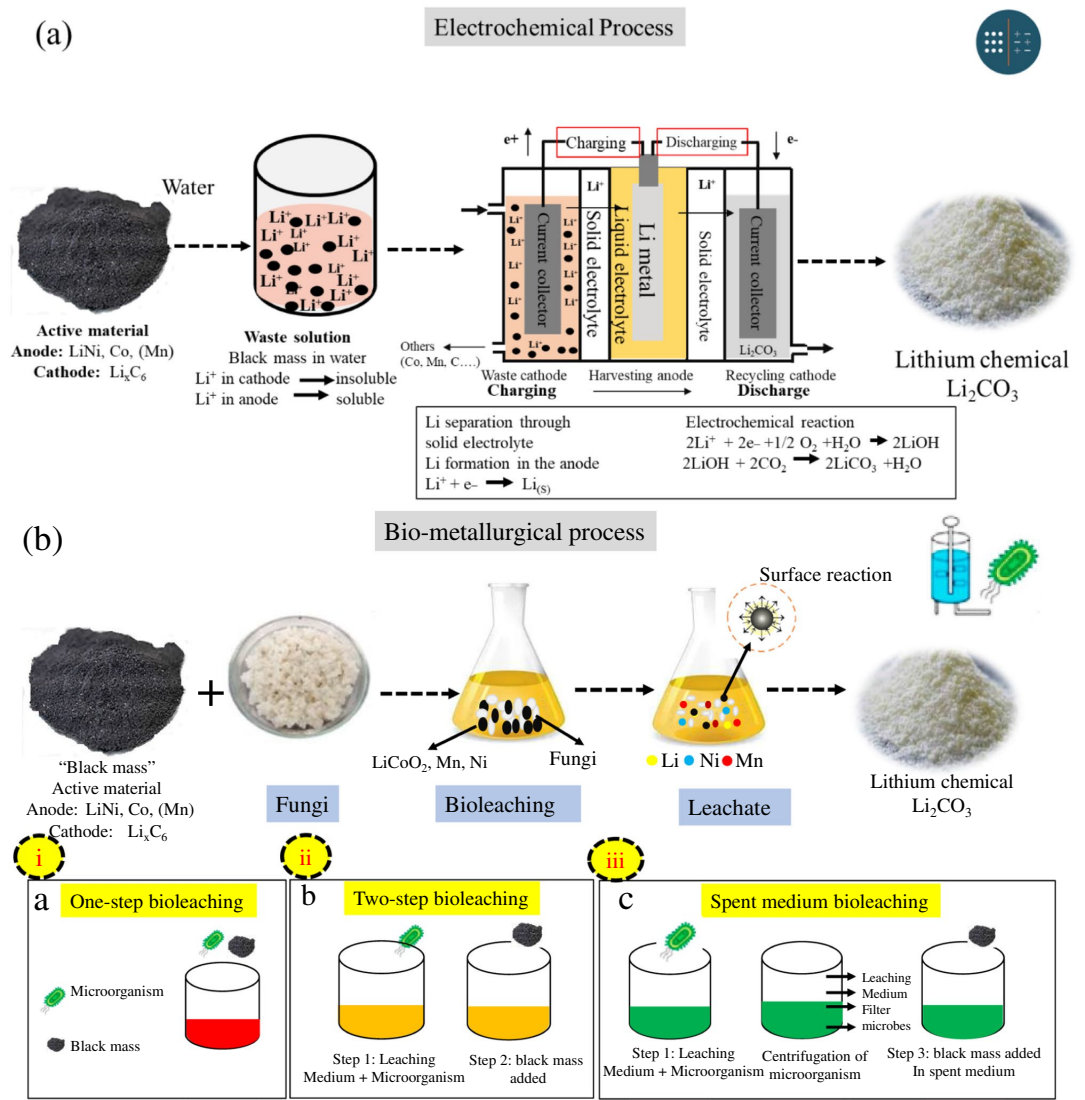
**a** Electrochemical extraction process for recovering lithium from pre-treated waste LIBs. **b** Different biometallurgical process (i) single-step separation, while (ii) shows the (a) one-step bioleaching, (b) two-step bioleaching, and (c) spent medium bioleaching for recovery of Li and other metals from black mass

Advanced research tackles this problem and developed the short steps to extract the LiOH directly. The side effect is extracting Li first at the very front of the chain, and this gives more flexibility in the building of value chain. This is what processes look like in Fig. 9a, (ii) which are divided into two parts. In the first step, mobilize (active) Li in the black mass while in the second step, leach the Li selectively in its format of LiOH. What remains is a black mass containing everything that was as before minus Li. This black mass can then be put into the hydrometallurgical plant for further isolation of Co, Ni, and other metals. Due to this direct LiOH extraction process, sodium sulfate (Na_2_SO_4_) formation is avoided completely and saves the additional steps required to turn Li_2_CO_3_ into LiOH. By advanced hydrometallurgical techniques, researchers and companies can reduce the carbon footprint beside the low proce of the technique (Bae and Kim 2021).

2.2.2.2.2. Pyrometallurgical process: Lithium, cobalt, nickel, and copper recovery only, graphite and solvents burned, high energy requirement (1500 °C), but the loss of lithium and hazardous gases toxic-waste by-product also increase the carbon footprint (Roy et al. 2021a).

Figure 9 b shows the overall process of recovery of Li_2_CO_3_; firstly, black mass is calcined at a > 700 °C process, then heated by putting it into the water where lithium (Li_2_CO_3_) is subsequently dissolved in the water via water leaching on the calcinated powder while metal oxide does not break down in water. The reactions take place in the cathode and anode (Wang et al. 2021), which makes Li soluble in water and allows organic material to be removed by evaporation at high temperatures, then lithium is recycled from the aqueous solution. The separation of the aqueous solution and the undissolved metal oxide is to create a Li_2_CO_3_ solution, and the subsequent water evaporation generated Li_2_CO_3_. This technique is straightforward and can handle a lot of waste lithium. The reactions that occur are shown in Eqs. (1) to (5):

2 $$C+12LiM{O}_{2}\to 6{Li}_{2}O+4{M}_{3}{O}_{4}+C{O}_{2}$$

3 $$C+4LiM{O}_{2}\to 2{Li}_{2}O+4MO+C{O}_{2}$$

4 $$2{M}_{3}{O}_{4}+C\to 6MO+C{O}_{2}$$

5 $${Li}_{2}O+C{O}_{2}\to {Li}_{2}C{O}_{3}$$

6 $$2MO+C\to 2M+C{O}_{2}$$

For lithium recycling, the major drawback in pyrometallurgy technique is that extra steps are necessary after calcination (Casals et al. 2019). This additional step typically involves melting the material in water or a solvent and breaking it. However, the less solubility of Li_2_CO_3_ (13 g/L^−1^) requires a huge quantity of solvent (Shin et al. 2023).

#### Electrochemical and biometallurgical methods

Electrochemical technology is an alternate for the extraction of Li and metals. The main benefits of this technology are that it does not harm the environment, is flexible, energy saving, safe to run, automated, and is cheaper than other methods. The black mass put into the water and separated Li by using Li-ion conductive ceramic solid electrolyte. The complete process of extracting of Li can be seen in Fig. 9a. When the black mass is put into the water, the Li in the cathode does not dissolve while Li in the anode dissolved in the water and formed the LiOH_(aq)_. In the next step, the solution is put into the electrochemical device and applied charge; by using the solid ceramic electrolyte, the dissolved and undissolved Li can be extracted. The dissolved Li^+^ can be extracted by oxygen transformation reaction (OTR), and the reaction process is shown in Eq. (7). The charging potential varies according to the cathode material in the wasted material:

7 $$2LiOH(aq)\to 2{Li}^{+}+{2e}^{-}+1/2{O}_{2}+{H}_{2}O$$

In cathode powder the undissolved Li^+^ can be separated by applying same technique as delithiation in cathode material (Eqs.(8) and (9)):

8 $$Li{MO}_{2}\to {Li}_{+}+{e}_{-}+{MO}_{2}(M=Co, Mn, Ni)$$

9 $$Li{FePO}_{4}\to {Li}_{+}+{e}_{-}+{FePO}_{4}$$

The aqueous solution converted a strong base due to the LiOH influence. OTR reaction occurs on charging lower 3.6 V and powerful base pH (pH > 11). In some conditions, Li^+^ is directly extracted from the cathode powder due to the delithiation potential of 3.5 V such as cathode powder of Li·Fe·PO_4_, which is lower than the voltage of OTR reaction. LiNi0.3Mn0.3Co0.3O_2_ (3.7 V), LiCoO_2_ (3.9 V), and LiMn_2_O_4_ (4.0 V), Li^+^ extraction from the solution, rather than from the cathode powder, because of delithiation potential is higher than that of the strong base OTR reaction (Leal et al. 2023). However, according to the features of the OTR reaction, the pH of the aqueous solution lowers during charging, and the operating voltage of the OTR reaction steadily increases. Li^+ ^can be separated by extraction in both powder and solution conditions (Bahaloo-Horeh et al. 2018).

Following the charging process, the separated Li^+^ ions start to travel another solid electrolyte and start a new reaction with H_2_O and electron (2e^−^) and form an aqueous solution of LiOH which can be expressed in Eq. 10. This reaction is called oxygen reduction reaction (ORR). Li_2_CO_3_ is formed when LiOH reacted with CO_2_ because it is a very strong base and is highly reactive with CO_2_ during the reaction. The formation process of Li_2_CO_3_ can be seen in Eq. 11:

10 $$2{Li}^{+}+ 2{e}^{-}+1/ {2O}_{2}+{H}_{2}O \to 2LiOH$$

11 $$2LiOH + {2CO}_{2}\to {2Li}_{2}{CO}_{3}+{H}_{2}O$$

Electrochemical extraction allows Li_2_CO_3_ powder to be obtained without the requirement for drying or precipitation, because Li_2_CO_3_ powder precipitates spontaneously when the system is discharged continuously. When a discharge happens, the level of Li_2_CO_3_ in the water progressively rises. Furthermore, because of the ORR, H_2_O is consumed, and the concentration of Li_2_CO_3_ rises relative to the reduction in solvent amount. This causes Li_2_CO_3_ to naturally precipitate (Roy et al. 2021b).

Bio-hydrometallurgy technology is an alternative strategy for removing and recovering precious metals over current treatment technologies (Horeh et al. 2016). This method is more feasible than traditional ones since it can offer a greater recovery yield even at low metal concentrations. The bioleaching process can function with a simpler setup, less energy and water use, and less toxic byproducts. Currently, this technology does not function at industrial scale, due to lowest technology keenness level and slow kinetics process.

In the bio-metallurgical process, bioleaching employs microorganisms such as fungus and chemolithotrophic and acidophilic bacteria (microbial metabolites) as leaching agents employing ferrous ion and sulfur as the source of energy to form metabolites in the leaching medium in order to recover Li and precious metals (Moazzam et al. 2021). However, compared to other traditional processes, bioleaching offers a number of advantages including higher recovery, simplicity, cost-effectiveness, and reduced energy consumption procedure without the requirement for severe environmental conditions or specialized industrial equipment (Roy et al. 2021b).

Figure 9 b shows three (i), (ii), and (iii) fundamental solid waste bioleaching techniques: (a) *one-step bioleaching*: The bioleaching procedure is finished in a single phase. Microorganisms and solid waste are added to the medium simultaneously, and the bioleaching process is carried out as the microorganisms are being cultivated. (b) Bioleaching has two steps: Two phases comprise bioleaching where without solid waste, bacteria achieve logarithmic phase, and solid waste is introduced to start bioleaching, and (c) spent-medium bioleaching: without solid waste, organisms can grow and make leaching agents after centrifuging and filtering the mixture, and solid waste is added to the cell-free medium to start the bioleaching process.

Two-step bioleaching yields more than one step. Compared to one-step and two-step bioleaching, wasted medium bioleaching is faster and easier. Because leaching and metabolite production are separated, both chemical and biological processes can be improved on their own. Higher temperatures and lower pH can increase metal recovery from black matter (Moazzam et al. 2021). The spent-medium approach requires extra bioreactor tanks due to microbial fermentation and two-stage use of metabolites.

#### Economic and environmental pollution issues

The EV batteries present both economic and environmental pollution challenges. Economically, the disposal and treatment of wasted EV batteries can be costly, and inefficient processes can lead to resource wastage (Efremova et al. 2022). Environmentally, improper disposal or treatment can result in the release of hazardous substances into the environment, including heavy metals and toxic chemicals, posing risks to ecosystems and human health (Koh et al. 2021). However, Table 1 provides a concise overview of the economic and environmental aspects, as well as the advantages and disadvantages of each treatment method for wasted EV batteries.

**Table 1:** A concise overview of the economic and environmental aspects, as well as the advantages and disadvantages of each treatment method for wasted EVs batteries

Treatment method	Economic issues	Environmental issues	Advantages	Disadvantages
Hydrometallurgical method	Costly treatment and disposal, resource wastage	Release of toxic substances into water, water usage, low temperature	Selective recovery of metals, suitable for low-grade ores	Requires significant water usage, generates toxic wastewater
Pyrometallurgical method	High initial investment, resource wastage	Air pollution (e.g., GHGs, sulfur dioxide emissions), energy usage	High metal recovery rates, effective for large-scale	Energy-intensive, air pollution, requires careful by-product management
Electrochemical method	High initial investment, operating costs	Potential pollution from chemical usage; energy usage	High-purity metal recovery, energy-efficient, use of renewable energy	High initial investment, operating costs, limited applicability
Bio-hydrometallurgical method	Moderate initial investment, potential for lower operating costs	Minimal pollution if properly managed, low energy usage	Environmentally friendly, operates under mild conditions	Slow process kinetics, requires careful environmental control, by-product managing

Overall, each treatment method for wasted EV batteries comes with its own set of advantages and disadvantages, impacting both economic viability and environmental sustainability. The selection of the most appropriate method depends on factors such as the composition of the batteries, scale of operation, environmental regulations, and economic considerations. Effective management of wasted EV batteries is essential to minimize pollution and maximize resource recovery in a sustainable manner (Zhang et al. 2020).

### S-curve analysis of treatment technologies

The investigating bibliometric data through an S-curve analysis provides a structured framework for identifying growth pattern, understanding the dynamics of technology adoption rate over time, and trend of future trajectory of technologies based on past data. This predictive capability is valuable for researchers, policymakers, and industry stakeholders seeking insights into the future trajectory of treatment technologies for lithium-ion batteries, as well as informed strategic decisions regarding research funding, policy interventions, and investment in scaling up promising technologies.

**Fig. 10:**
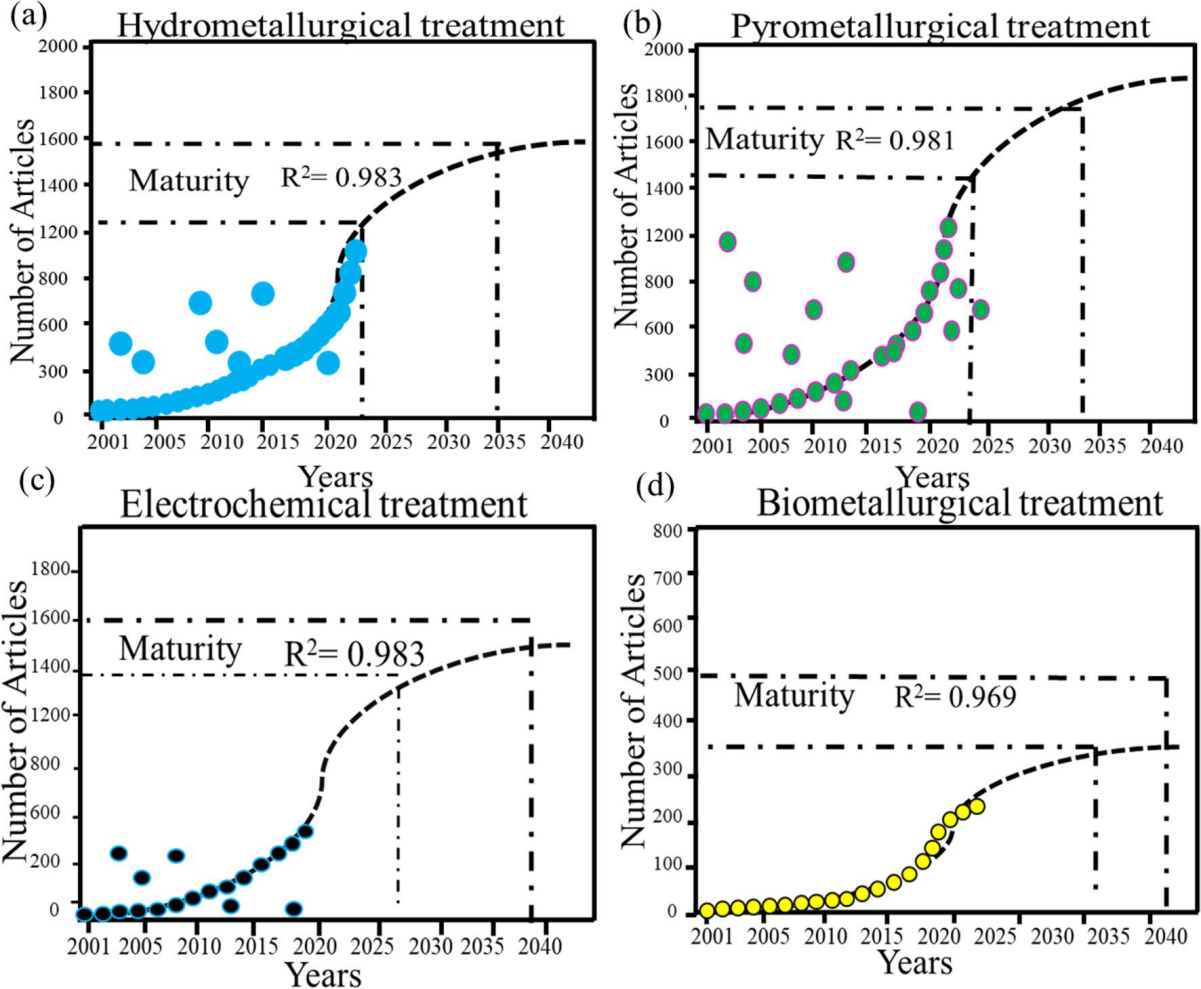
The S-curves analysis of hydrometallurgical (**a**), pyrometallurgical (**b**), electrochemical (**c**), and biometallurgical (**d**) technologies

The S-curve analysis is a common tool used in various fields to assess the maturity and adoption of technologies over time based on published research. In the context of metallurgy, different processes may enter maturity stages at different times based on factors such as technological advancements, market demand, and regulatory changes (Brückner et al. 2020). This research analysis provides a specific timeline based on published articles (Yun et al. 2018) to simulate the overall trends of treatment methods. This research applied the S-curve to further explore specific treatment technology tendency; we subdivided the recycling technologies into the four treatment categories like hydrometallurgy, pyrometallurgy, electrochemical, and biometallurgy (Hua and Dong 2022). Their development trends are shown in Fig. 10. The S-curves derived from the articles demonstrated the effectiveness of all treatment methods entered into the maturity stage by 2023, 2024, 2026, and 2031 respectively. S-curve maturity means any project or research that pass through the emerging, growing, and maturity stage. At this point, the technology has achieved widespread acceptance, and adoption rates begin to level off. The market becomes saturated, meaning that most potential customers who are willing to adopt the technology have already done so (Lai et al. 2022).

Figure 10 a shows that the hydrometallurgical treatment technology was closer to the saturation stage that would enter the maturity stage around 2023, and in Fig. 10b, pyrometallurgy would enter the maturity stage around by 2024. Differently, electrochemical (Fig. 10c) and biometallurgical (Fig. 10d) treatment technology would have a much longer time before reaching the saturation stage and have a greater potential for innovation and development. The total published articles of the hydrometallurgical treatment method were 1361, higher than pyrometallurgical (1114) and electrochemical (969), and biometallurgical (743) till 2021. Both S-curve and publication output indicated the popularity of hydrometallurgical treatment method because processes may have reached maturity due to decades of research and development. On the other hand, biometallurgical technology is showing very slow as compared to the other three technologies, because this emerging technology is nonfunctional (open loop) at industrial level because of the slow process of extracting metals.

The hydrometallurgical treatment method had the advantages of high efficiency, low cost, and convenient operation and has played a crucial role in wasted LIB recycling (Bahaloo-Horeh et al. 2018), with the researchers’ much attention and the huge number of studies, the maturity of hydrometallurgical treatment technologies has improved rapidly. On the other hand, pyrometallurgical processes involve high-temperature reactions to extract metals from ores and concentrates. Some traditional pyrometallurgical techniques, such as smelting and roasting, have been refined over centuries and are considered mature technologies. However, advancements in areas like furnace design, process control, and environmental considerations may still lead to innovations within this field (Atia et al. 2019). Follow-on the total number of publications and value of *R*^2^ = 0.981 in Fig. 10c, the pyrometallurgical method is little after hydrometallurgical technological methods. Figure 10 c, electrochemical treatment method, has 969 publications and lies in growing stage. In this stage, the technology experiences increasing adoption and market penetration. Demand for the technology grows as awareness spreads, and early adopters start to embrace it. Growth rates are typically high during this phase as the technology gains traction and captures market share (Horeh et al. 2016). Investments in scaling up production, expanding distribution channels, and improving efficiency are common in the growth stage. Competition may intensify as more players enter the market to capitalize on the opportunity.

Lastly, bio-hydrometallurgy is a relatively newer field that involves using microorganisms to facilitate metal extraction from ores and concentrates. Bio-hydrometallurgical processes have gained attention for their potential environmental benefits and low energy requirements. While still in a growing stage which can be accessed through Fig. 10d, there is limited amount of (743) publication till 2021 as compared to the other three technologies. During this emerging stage, adoption is typically low, and the technology may still be undergoing development and refinement. This emerging technology is facing uncertainty and may require significant investment in research, development, and market education. Moreover, there is potential for rapid growth if the technology proves to be viable and addresses a significant need or market opportunity. In terms of specific technologies, compared with pyrometallurgical and electrochemical treatment methods, more research on the biometallurgical treatment method is necessary. Overall, so far, through the results of Table 1 and Fig. 10, the hydrometallurgy offers a more environmentally friendly and economically viable approach to recycling lithium-ion batteries compared to pyrometallurgy and other technologies. Additionally, ongoing research and development efforts aim to improve the efficiency, sustainability, and economics of battery recycling processes (Jayaraman and Thottungal 2024).

## Future perspective

Scientometric analysis of wasted EVBs can be a useful tool to measure the performance of second life use and recycling practices, assess research gaps, and identify potential new research areas. As electric vehicles become increasingly popular, there is potential to expand bibliometric analysis to gain valuable insights into usage patterns, adoption rates, and other economic and environmental factors that could impact sustainability efforts. As a result of this research approach, researchers in the future will be able to apply artificial intelligence that is distributed across citation networks or artificial neural network to discover in the field of second life use and treatment methodologies of Li-ion wasted EVBs.

Besides this, there are some explicit guidance points for future research directions related to second-life usage and recycling methodologies of wasted EV batteries: In second-life usage, explore the novel applications and technologies for repurposing EV batteries beyond their initial automotive use. Investigate the feasibility and effectiveness of integrating EV batteries into renewable energy storage systems, grid stabilization solutions, or off-grid applications and potential for medical uses such as powering pacemakers, insulin pumps, and other medical devices. Conduct life cycle assessments to evaluate the environmental and economic impacts of second-life applications compared to traditional recycling approaches. Moreover, investigate policy frameworks, regulations, and incentives to encourage the adoption of second-life usage models and promote circular economy principles in the EV battery industry. This could dramatically reduce the need for alternative, non-renewable energy sources in these areas.

On the other hand, recycling methodologies develop innovative and efficient recycling processes that can recover valuable materials from wasted EV batteries whereas minimizing environmental impacts. Explore advanced separation and purification techniques to enhance the recovery rates of critical materials such as lithium, cobalt, and nickel from spent EV batteries. Investigate the scalability and commercial viability of emerging recycling technologies, such as hydrometallurgical, pyrometallurgical, electrochemical, and bio-hydrometallurgical methods. Moreover, assess the environmental footprint of different recycling methodologies, considering factors such as energy consumption, greenhouse gas emissions, and waste generation. Besides, explore synergies between EV battery recycling and other industries to develop integrated waste valorization strategies and resource recovery networks. Investigate the potential for decentralized or localized recycling facilities to reduce transportation costs and environmental burdens associated with battery waste management.

In the future, the cross-cutting themes can foster interdisciplinary collaborations between researchers, industry stakeholders, policymakers, and environmental organizations to address the complex challenges of second-life usage and EV battery recycling. Emphasize the importance of public awareness, education, and engagement initiatives to promote responsible consumer behavior, encourage battery reuse, and improve recycling rates. Prioritize research efforts that prioritize environmental sustainability, social equity, and economic viability throughout the entire EV battery lifecycle. Finally, foster international cooperation and knowledge sharing to accelerate technology development, standardization, and best practices in the field of EV battery reuse and recycling. By focusing on these research directions, scholars and practitioners can contribute to the advancement of sustainable solutions for managing wasted EV batteries and promoting the transition towards a circular economy in the electric mobility sector.

## Conclusion

In conclusion, a study analyzed 4810 relevant articles from SCI and SSCI Scopus databases by using VOSviewer, ArcGIS, Pajek, and Netdraw software. The scientometric analysis shows the growing interest of using the Li-ion batteries with development of EV industry as a resulting massive generating of batteries which need to manage through second life use and recycling methodologies. Subsequent exploring in second life applications and recycling methodologies in China, Germany, and the USA lead in research output, while institutions like Tsinghua University and Fraunhofer ISI are prominent in this field. In second life, stationary (solar, wind, and tidal energy storage, solar, wind, and tidal energy, long-distance houses and cabins, emergency lighting, e-bikes and rickshaw as well as trains which run for picnicking) is the way of using. By analyzing the co-occurrence, keywords and S-curve analysis, treatment methods (hydrometallurgy and pyrometallurgical, electrochemical, biometallurgical) were the hot research topics. Moreover, to classify the research potential from both academic research and practical application perspectives, both repurposing and recycling of Li-ion batteries offer significant economic and environmental advantages by maximizing resource utilization, reducing waste generation, and minimizing environmental impacts throughout the battery lifecycle.

Besides, the study analysis suggests that the first alternative for beneficial economic and environmental impacts is repurposing lithium-ion batteries for second-life applications. The second alternative involves recycling lithium-ion batteries to extract valuable materials. Lastly, the third alternative focuses on improving the design and manufacturing processes of lithium-ion batteries to enhance their recyclability and environmental sustainability. Finally, these findings are beneficial for academic researchers, industry professionals, government decision-makers, and anybody else concerned in this research area.
